# Increasing Trends on Intercostal Chest Tube Placement for Hepatic Hydrothorax Despite Negative Impact on Patient Outcomes: A National Inpatient Sample Database Analysis

**DOI:** 10.14309/ctg.0000000000000889

**Published:** 2025-07-11

**Authors:** Aishwarya Thakurdesai, Sheel Patel, Winston Dunn, Paul Kwo, Ashwani K. Singal

**Affiliations:** 1 University of Louisville Health Sciences Center, Louisville, Kentucky, USA;; 2 Kansas University Medical Center, Kansas City, Kansas, USA;; 3 Stanford University Medical Center, Stanford, California, USA;; 4 Trager Transplant Center at Jewish Hospital, Louisville, Kentucky, USA; 5 Robley Rex VA Medical Center, Louisville, Kentucky, USA.

**Keywords:** chest tube, empyema, pleural effusion

## Abstract

**INTRODUCTION:**

Hepatic hydrothorax (HH) significantly contributes to morbidity in decompensated cirrhosis. Intercostal chest tube (ICT) insertion is discouraged in HH management. We examined trends in ICT use and impact on outcomes in hospitalized HH patients.

**METHODS:**

A retrospective cohort study (October 2015–December 2019) was conducted using the National Inpatient Sample to identify HH hospitalizations among patients with decompensated cirrhosis. Propensity score matching compared patients who received ICT with those who did not. Outcomes included in-hospital mortality (IHM), length of stay (LOS), total charges (TC), and complications.

**RESULTS:**

Among 127,627 cirrhosis hospitalizations, 7,843 (6.2%) had HH. Compared with those without HH, these patients had longer LOS, higher TC, and more acute kidney injury and sepsis (*P* < 0.001). HH was not associated with increased IHM, but ICT and spontaneous bacterial empyema were, each conferring ∼1.5-fold higher odds. ICT was used in 1,312 HH cases (16.7%), with increasing use over time (*P* = 0.037). In a matched cohort of HH hospitalizations (1,277 with ICT; 2,554 without), ICT use was linked to higher IHM (11.6% vs 8.5%), longer LOS (14.6 vs 8.7 days), and greater TC ($196,000 vs $112,000). Complications were more frequent with ICT: acute kidney injury (45% vs 39%), sepsis (18% vs 10%), and spontaneous bacterial empyema (12.5% vs 2%) (*P* < 0.001). ICT use was associated with 44% higher odds of IHM, OR (95% confidence intervals): 1.44 (1.15–1.81).

**DISCUSSION:**

HH occurs in 6.2% of cirrhosis hospitalizations, with 1 of 6 receiving ICT. ICT use is increasing despite poorer outcomes and greater resource utilization. Studies targeted toward better patient selection and provider education are needed to mitigate ICT use in HH.

## INTRODUCTION

Hepatic hydrothorax (HH) is a rare but serious complication of cirrhosis that affects 5%–10% of patients, particularly those with decompensated liver disease (1). It is thought that HH involves the movement of ascitic fluid into the pleural cavity through small defects in the diaphragm. These openings are lined with a pleuroperitoneal membrane that may tear when intra-abdominal pressure increases, allowing fluid to flow into the low-pressure pleural space (2). HH is most often unilateral, predominantly affecting the right hemithorax (3). Diagnosis is established through pleural fluid analysis, which typically reveals a transudative effusion with a serum-to-pleural fluid albumin gradient exceeding 1.1 g/dL (3). Patients who develop HH are associated with a poor prognosis, facing a significant risk of both morbidity and mortality (4). The primary goal in the treatment of HH is centered on addressing the excess fluid buildup by eliminating and preventing the recurrence of ascites. Initial management focuses on sodium restriction and initiation of diuretics (5). It is estimated that 21%–26% of patients continue to experience dyspnea and respiratory compromise even after optimization of dietary salt intake and diuretics (6). These patients are categorized as having refractory HH, a condition that is challenging to manage clinically (7). In such cases, patients should be evaluated for a transjugular intrahepatic portosystemic shunt (TIPS), which alleviates and resolves symptoms in 59%–81% of cases (8). TIPS can serve as a bridge to liver transplantation (LT) or definitive treatment in patients ineligible for LT (9).

Select patients who are not candidates for TIPS or LT can be treated with thoracentesis, indwelling pleural catheters, or intercostal chest tubes (ICTs) (5). While thoracentesis can provide prompt symptomatic relief, fluid reaccumulates quickly, necessitating repeated thoracentesis (10). In these cases, patients may choose to have an indwelling pleural catheter placed. Although thoracentesis and indwelling pleural catheters are commonly used to manage refractory HH, providers often resort to intercostal chest tube (ICT) placement. However, societies recommend against the use of ICT with observational data showing association with risks and negative patient outcomes (11).

We performed this study to examine the trends in the use of ICT in the management of HH. We also examined the association of ICT with patient outcomes (mortality and liver complications) and hospital resources use among hospitalized patients with decompensated cirrhosis in the United States

## METHODS

### Database

The National Inpatient Sample (NIS) database was used for the study. With a relatively stable trend in cirrhosis etiology over the past 5 years and the use of common *International Classification of Diseases, Tenth Revision* (*ICD-10*) diagnosis and procedure codes, the study population included hospitalizations between October 2015 and December 2019. The NIS is the most extensive all-payer database for hospital discharges in the United States It is managed under the Healthcare Cost and Utilization Project by the Agency for Healthcare Research and Quality. This database provides anonymized details about hospitalizations, including patient demographics, admission and discharge information, diagnoses, procedures, comorbidities, outcomes, and hospital charges. The hospitals included in the sample are selected based on factors such as size, geographic location (urban or rural), region, ownership type, and teaching status.

### Study population

Hospitalizations due to cirrhosis and HH were identified using *ICD-10* code (K70.11) at discharge. Admissions of individuals aged younger than 18 years and those without a diagnosis of liver cirrhosis were excluded.

Baseline characteristics were compared between hospitalizations with vs without HH for patient demographics (age, sex, race), etiology of cirrhosis, comorbidities (obesity and diabetes mellitus), insurance status (Medicare, Medicaid, private), hospital location and teaching status (rural, urban nonteaching, and urban teaching), and hospital size (small, medium, large) based on the number of beds.

Hospitalizations with HH were stratified based on the use of ICT during the hospitalization using a discharge procedure code 0B960Z2. Trends in the use of ICT were examined. HH hospitalizations requiring ICT were compared with those who did not undergo ICT placement for all the baseline characteristics listed above.

### Creating a matched cohort

A logistic regression model was developed to examine baseline characteristics associated with the use of ICT for the management of HH. Each hospitalization with HH receiving ICT was propensity score matched with 2 hospitalizations not managed with ICT. Variables associated with ICT use on logistic regression model and those clinically relevant were used for the matching process.

### Outcomes

The matched cohort was examined for the study outcomes:

Primary outcome was in-hospital mortality (IHM).

Secondary outcomes were the following: (i) cirrhosis complications (acute kidney injury [AKI], hepatorenal syndrome [HRS], esophageal variceal hemorrhage, hepatic encephalopathy, sepsis, spontaneous bacterial empyema), (ii) use of hospital resources (thoracentesis, liver transplant, palliative care consultation), and (iii) economic burden (length of stay in days and total hospital charges in USD). Liver-related complications were identified using *ICD-10* disease codes, and hospital resources were identified using *ICD-10* procedure codes. Conditional logistic regression model was developed to examine the independent association of ICT use and other variables with IHM.

### Data and statistical analysis

The Armitage trend test was used for statistical analysis. Baseline characteristics were compared using χ^2^ and ANOVA tests for categorical and continuous variables, respectively. In propensity score matching, variables were considered matched if the mean difference between the 2 groups (ICT vs no ICT) was less than 0.1. Odds ratios with 95% confidence intervals (CIs) were reported on logistic regression analyses.

SAS software 9.4 (SAS Institute, Cary, NC) was used for statistical analyses. A *P*-value of <0.05 was considered statistically significant.

## RESULTS

### Baseline characteristics of patients with and without hydrothorax

Of 754,245 hospitalizations between October 2015 and December 2019, 7,843 (1.04%) had HH. Overtime, trend on hospitalizations with HH was unchanged (*P* = 0.151 and *P* = 0.832, respectively).

Among hospitalizations with vs without HH, patients were younger with a lower proportion of women; obesity and diabetes; were more likely to be African American; and receive care at nonteaching hospitals in moderate-sized hospitals (Table 1).

**Table 1.** Baseline characteristics comparing cirrhosis hospitalizations with vs without discharge diagnosis of hydrothorax

	Variable	No hydrothorax (N = 119,763)​	Hydrothorax (N = 7,843)​	*P*​
	Demographics			
	Mean age, SD​	63.3–12.7	59.4–11.8	<0.001
	Female sex	56,088 (46.8)	3,540 (45.1)	0.004
	Whites	76,791 (62)	5,274 (69.5)	
Race	African Americans	13,131 (10.6)	386 (5.1)	<0.001​
	Hispanics​	18,016 (14.5)	1,368 (18)	​
Insurance type	Medicare​	68,178 (53.5)	3,422 (43.7)	
	Medicaid​	20,973 (17.5)	1925 (24.6)	<0.001​
	Private​	23,179 (19.4)	1950 (24.5)	​
	Obesity	18,108 (15.1)	1,052 (13.4)	<0.001​
	Diabetes mellitus	54,315 (45.3)	2,594 (33.1)	<0.001​
	Rural	7,883 (6.6)​	255 (3.3)​	​
Hospital type	Nonteaching urban​	23,553 (19.7)​	1,054 (13.4)​	<0.001​
	Teaching urban​	88,348 (73.8)​	6,534 (83.3)​	​
	Small hospital​	19,667 (16.4)​	985 (12.6)​	​
Hospital size	Moderate hospital​	32,470 (27.1)​	1857 (23.7)​	<0.001​
	Large hospitals	67,647 (56.5)​	5,001 (63.8)​	​
	Weekend admissions​	27,191 (22.7)​	1845 (23.5)​	0.092​

### Outcomes HH vs no HH hospitalizations

**Figure 1.**
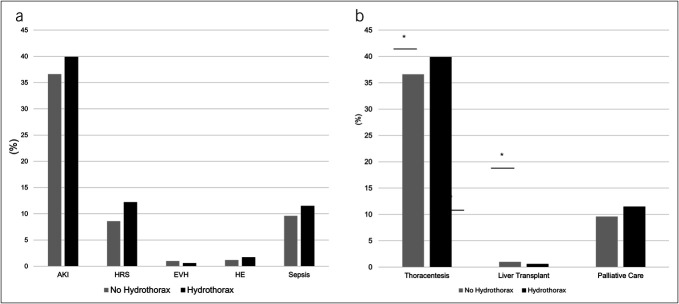
(**a**) Comparison of complication rates between hospitalizations with vs without hydrothorax. *Denotes *P* value < 0.001, indicating statistically significant difference. (**b**) Comparison of utilization rates on hospital resources between hospitalizations with vs without hydrothorax. *Denotes *P* value < 0.05, indicating statistically significant difference. AKI, acute kidney injury; HRS, hepatorenal syndrome; EVH, esophageal variceal hemorrhage; HE; hepatic encephalopathy; TIPS, transjugular intrahepatic portosystemic shunt.

Hospitalizations with vs without HH had similar IHM (9.1 vs 8.4%, *P* = 0.10). However, liver disease complication rates were higher for AKI (39.9% vs 36.6%), HRS (12.2% vs 8.6%), HE (1.7% vs 1.2%), and sepsis, *P* < 0.001 for all. The proportion of hospitalizations complicated by esophageal variceal hemorrhage was lower (0.6 vs 1.0%, *P* < 0.001), Figure 1A. Similarly, hospitalizations with HH required more hospital resources such as thoracentesis (42.4% vs 31.0%, *P* < 0.001), LT (3.5% vs 1.7%, *P* < 0.001), and palliative care consult (11.3 vs 10.3%), *P* < 0.001 for all, Figure 1B. Only 2 hospitalizations required TIPS placement, both in the no HH group. Furthermore, hospitalizations with vs without HH had higher mean length of stay (9.8 ± 11.4 vs 7.4 ± 8.6 days) and total charges (126,692 ± 222,241 vs 89,179 ± 168,153 USD), *P* < 0.001 for both.

### Variables associated with IHM

In a logistic regression multivariable model for the outcome of IHM, cirrhosis due to alcohol-associated liver disease (ALD) vs chronic viral hepatitis (CVH), patient's age, Asian or other vs White race, Medicaid or other vs Medicare payment source, and hospitalization in urban teaching and large hospitals were associated with IHM. By contrast, NASH vs CVH etiology of cirrhosis was protective for IHM. Other variables associated with IHM were ICT use and SBE with 50% and 48% higher odds for IHM (Table 2).

**Table 2.** Variables associated with in-hospital mortality in hospitalizations with hydrothorax

​Variable	Odds ratio (95% CI)	*P*​
Hydrothorax​	0.95 (0.84–1.07)​	0.424​
Calendar year​	0.97 (0.93–1.00)​	0.073​
Patient's age in years​	1.02 (1.01–1.02)​	<0.001​
Female​ sex	0.94 (0.86–1.04)​	0.236​
African American vs White race​	1.16 (0.99–1.36)​	0.059​
Hispanic vs White race​	0.95 (0.83–1.09)​	0.477​
Asian or other vs White race​	1.24 (1.05–1.48)​	0.014​
Alcohol-associated liver disease vs chronic viral hepatitis​ (CVH)	1.32 (1.10–1.58)​	0.003​
Metabolic dysfunction-associated steatohepatitis vs CVH	0.80 (0.66–0.98)​	0.030​
Medicaid vs Medicare insurance	1.16 (1.01–1.33)​	0.033​
Private vs Medicare insurance	1.09 (0.96–1.24)​	0.182​
Other vs Medicare insurance	1.27 (1.05–1.53)​	0.013​
Urban nonteaching vs rural​ hospital	1.10 (0.85–1.42)​	0.477​
Urban teaching vs rural​ hospital	1.30 (10.3–1.64)​	0.026​
Medium vs small size hospital​	1.07 (0.92–1.26)​	0.378​
Large vs small size hospital​	1.22 (1.06–1.40)​	0.006​
Intercostal chest tube placement	1.50 (1.25–1.79)​	<0.001​
Spontaneous bacterial empyema	1.48 (1.08–2.04)​	0.015​

CI, confidence interval; CVH, chronic viral hepatitis.

### ICT use and trends

Of 7,843 hospitalizations with HH, 1,312 (16.7%) received ICT for the management of HH. Annual proportion of hospitalizations with HH receiving ICT between 2015 and 2019 were 13.8% (64 of 164), 15.1% (261 of 1728), 17.4% (299 of 1719), 18.3% (350 of 1915), and 16.8% (338 of 2017), respectively, Trend *P* = 0.037.

### Baseline characteristics (ICT vs no ICT use)

Hospitalizations with vs without ICT use were older, with lower proportion of women and weekend admissions. A significantly higher proportion of patients without ICT placement was obese, as compared with those with ICT placement (13.9% vs 11.2%, *P* = 0.01). There was no difference on race, insurance type, comorbid diabetes mellitus, type and size of admitting hospital, and liver disease etiology (Table 3).

**Table 3.** Baseline characteristics comparing with vs without ICT placement and variables associated with ICT placement in a cohort of cirrhosis hospitalizations and hydrothorax

	Variable	No ICT (N = 6,531)	ICT (N = 1,312)	*P*	Odds ratio (95% CI)
	Mean age, SD​	59.3–11.8​	60.2–11.6​	0.011​	1.01 (1.00–1.01)
	Female sex	2,985 (45.7)​	555 (42.3)​	0.024​	0.88 (0.77–0.99)
Race	Whites​	4,396 (69.6)​	878 (68.8)​	​	Ref.
	African Americans	321 (5.1)​	65 (5.1)​	0.803​	1.01 (0.77–1.34)
	Hispanics​	1,137 (18)​	231 (18.1)​	​	1.01 (0.86–1.19)
	Obesity	905 (13.9)​	147 (11.2)​	0.010​	
	Diabetes mellitus	2,174 (33.3)​	420 (32)​	0.370​	
Insurance type	Medicare​	2,824 (43.3)​	598 (45.6)​	​	Ref.
	Medicaid​	1,598 (24.5)​	327 (24.9)​	0.186​	1.07 (0.89–1.28)
	Private​	1,654 (25.4)​	296 (22.3)​	​	0.90 (0.76–1.06)
	Academic hospital ​	5,424 (83.1)​	1,110 (84.6)​	0.169​	1.13 (0.95–1.33)
	Large hospital	4,171 (63.9)​	830 (63.3)​	0.679​	0.99 (0.87–1.12)
	ALD​	2,642 (40.5)​	530 (40.4)​	​	1.17 (0.95–1.44)
Cirrhosis etiology	Chronic viral hepatitis​	832 (12.7)​	143 (10.9)​	0.057​	Ref.
	MASH​	998 (15.3)​	184 (14)​	​	1.03 (0.80–1.33)
	Weekend admissions​	1,567 (24)​	278 (21.2)​	0.029​	1.02 (0.85–1.15)
	Calendar year				1.05 (1.00–1.11)

ALD, alcohol-associated liver disease; CI, confidence interval; ICT, intercostal chest tube.

In a logistic regression multivariable model, variables associated with ICT use were age of the patient and cirrhosis etiology other than ALD, MASH, or CVH. On the other hand, women had 12% lower odds of ICT placement as compared with men. Ethnicity, insurance type, and hospital type or size were not associated with ICT placement (Table 3).

### Propensity-matched cohort (ICT vs no ICT use)

A total of 1,277 of 1,312 hospitalizations with ICT placement for the management of HH could be matched (1:2) with 2,554 HH hospitalizations without the use of ICT during the hospitalization. Variables included in the matching process were age, sex, race, cirrhosis etiology, academic status (urban teaching), and size of hospital (Table 1). The developed cohort matched for all the variables (Table 4). This matched cohort was examined for the study outcomes.

**Table 4.** Baseline characteristics comparing with vs without ICT placement in a propensity matched cohort of cirrhosis hospitalizations and hydrothorax

	Variable	No ICT (N = 2,554)​	ICT (N = 1,277)​	*P*
	Mean age, SD	60.1–11.6​	60.2–11.7​	0.699​
	Female sex	1,080 (42.3)​	544 (42.6)​	0.853​
	Whites​	1793 (70.2)​	878 (68.8)​	​
Race	African Americans	127 (5)​	65 (5.1)​	0.472​
	Hispanics​	463 (18.1)​	231 (18.1)​	​
	Obesity	287 (11.2)​	143 (11.2)​	0.97​
	Diabetes mellitus	869 (34)​	407 (31.9)​	0.183​
Insurance type	Alcohol-associated liver disease	1,044 (40.9)	518 (40.6)	0.853
	Medicare​ insurance	1,146 (44.9)​	587 (46)​	​
	Medicaid​ insurance	614 (24.1)​	319 (25)​	0.251​
	Private​ insurance	637 (25)​	283 (22.2)​	​
	Academic hospital ​status	2,101 (82.3)​	1,078 (84.4)​	0.095​
	Large size hospital	1,613 (63.2)​	801 (62.7)​	0.795​
	Weekend admissions​	530 (20.8)​	271 (21.2)​	0.736​

ICT, intercostal chest tube.

### Primary outcome

#### In-hospital mortality

**Figure 2.**
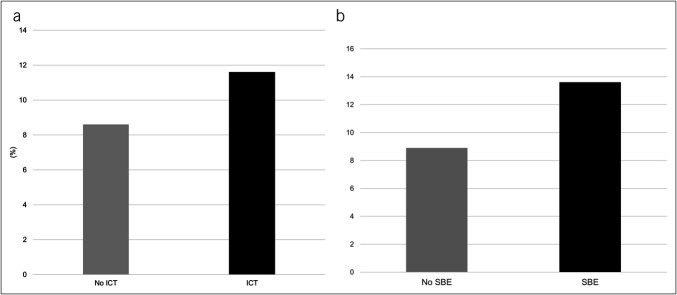
(**a**) Comparison of IHM between hospitalizations with vs without ICT placement in hospitalized patients with hepatic hydrothorax. *Denotes *P*-value = 0.004. (**b**) Comparison of IHM between hospitalizations with vs without spontaneous bacterial empyema in hospitalized patients with hepatic hydrothorax who received ICT. *Denotes *P*-value < 0.001, indicating statistically significant difference. ICT, intercostal chest tube; IHM, in-hospital mortality; SBE, spontaneous bacterial empyema.

IHM was higher for hospitalizations requiring ICT placement (11.6% vs 8.5%, *P* = 0.002), Figure 2A. This was further higher among hospitalizations with ICT with vs without SBE (13.6% vs 8.9%, *P* = 0.004), Figure 2B.

A conditional logistic regression model controlled for calendar year to examine variables associated with IHM. ALD vs CVH etiology of cirrhosis, age of the patient, and weekend admissions were associated with IHM. By contrast, hospitalizations with diabetes mellitus comorbidity were associated with 39% reduced odds for IHM. Other variables such as sex, race, comorbid obesity, type of insurance, and admitting hospital type and size were not associated with IHM. After controlling for all variables (Table 4), ICT placement was associated with 44% higher odds of dying during the hospitalization as compared with hospitalizations with HH without an ICT placement, OR 1.44 (95% CI 1.15–1.81), *P* < 0.001.

### Secondary outcomes

#### Liver disease complications

**Figure 3.**
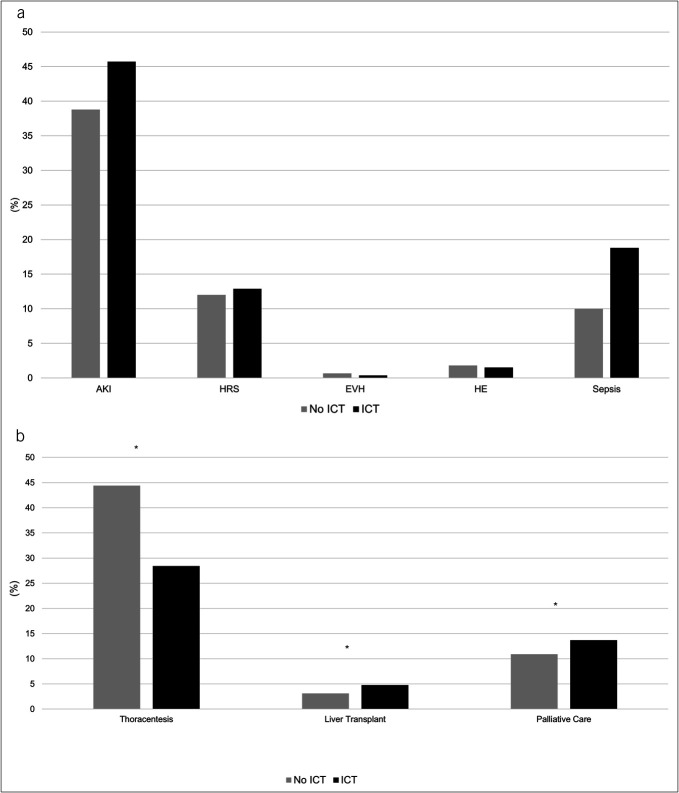
(**a**) Comparison of complication rates between hospitalizations, with vs without ICT placement. *Denotes *P*-value < 0.001, indicating statistically significant difference. (**b**) Comparison of utilization of hospital resources between hospitalizations with vs without ICT placement. *Denotes *P*-value < 0.05, indicating statistically significant difference. AKI, acute kidney injury; EVH, esophageal variceal hemorrhage; HE; hepatic encephalopathy; HRS, hepatorenal syndrome; ICT, intercostal chest tube.

There was an association of ICT vs no ICT use for AKI (45% vs 38.5%, *P* < 0.001), sepsis (18.3% vs 9.8%, *P* < 0.001), and SBE (12.5% vs 2.2%, *P* < 0.001). There were no differences in other liver disease complications, Figure 3A.

#### Use of hospital resources

Hospitalizations with ICT placement more often required LT (4.8% vs 3.1%, *P* = 0.007) and palliative care consult (13.7% vs 10.9%, *P* = 0.01). By contrast, as expected, there was a lower need for thoracentesis in hospitalizations using ICT (28.4% vs 44.4%, *P* < 0.001), Figure 3B.

#### Economic burden

Hospitalizations requiring ICT placement resulted in a substantially greater economic burden as compared with hospitalizations without ICT, both in the overall and matched cohorts. LOS was longer for ICT hospitalizations (Overall: 14.8 ± 13.6 days vs 8.8 ± 10.7 days; Matched: 14.6 ± 13.1 days vs 8.7 ± 10.7 days; *P* < 0.001 for both), as were total charges (Overall: 197,517 ± 285,419 USD vs 112,426 ± 204,275 USD; Matched: 195,618 ± 282,950 USD vs 111,737 ± 222,486 USD; *P* < 0.001 for both).

## DISCUSSION

Our study using the NIS database on hospitalizations for cirrhosis demonstrates increasing ICT placement in the management of HH. Furthermore, ICT placement is associated with patient morbidity with liver disease complications, hospital resource utilization, and patient mortality during hospitalization.

Higher patient morbidity with liver disease complications and infection rate as seen in our study has been shown earlier (12,13). Furthermore, association of ICT placement with development of sepsis, SBE, and AKI has also been shown earlier (14). This is because ICT placement is easy for the providers and maybe the patient given no need for repeat thoracentesis. However, this procedure does not reverse the pathophysiology of HH.

Our study found that patients who underwent ICT placement had a significantly longer hospital stay and incurred nearly 2 folds higher hospital charges compared with those without ICT. These findings are also in line with prior studies that have reported prolonged hospitalizations and increased healthcare costs associated with ICT placement in HH patients (15).

One of the most notable complications observed in our study was the high incidence of SBE among patients with ICT placement. The placement of a chest tube creates a direct conduit for bacterial translocation, predisposing patients to life-threatening infections. Prior studies have shown that chest tube placement in cirrhotic patients is associated with an increased risk of empyema and sepsis, leading to significantly higher mortality rates (3,13). These findings are further corroborated by our study, as we observed a nearly 50% increase in the odds of IHM among patients who underwent ICT placement (odds ratios 1.44, 95% CI 1.15–1.81, *P* < 0.001).

Repeated thoracentesis is an effective low-risk intervention for HH, as it allows for controlled fluid removal and decreases the risk of infection and hemodynamic instability (16). However, it requires repeated procedures, which can be cumbersome for patients. Transjugular intrahepatic portosystemic shunt placement is an option for patients with refractory HH to first-line treatment of sodium control, diuretics, and thoracentesis (10). TIPS can serve as a bridge to LT and has been shown to improve symptoms in 59–81% of patients with refractory HH (17,18). Despite guideline recommendations, the observed TIPS utilization in our study cohort was exceedingly low. This may reflect contraindications, delayed consideration, or potential coding omissions in administrative data sets. These finding underscores real-world gaps in care for patients with refractory HH.

However, when TIPS is contraindicated for some reason and patients are not eligible for LT, it is better to place a pigtail indwelling pleural catheter as a palliative measure. Although we were unable to separately identify indwelling pleural catheters from ICTs due to coding limitations, studies have shown that IPCs are associated with lower rates of infection and fewer hospitalizations compared with ICT placement (15,19). A systematic review demonstrated that IPCs provide effective symptomatic relief in HH patients while posing a low risk of complications (19). Although IPCs are not without risk, especially in the presence of coagulopathy, it provides a safer alternative to chest tubes in carefully selected patients. Future studies should address their utilization and comparative outcomes in HH.

Our study showed a concerning and novel finding that despite negative association of ICT with patient outcomes, its use has been increasing overtime. One possible explanation for the rising trend in ICT placement may be due to a lack of awareness among healthcare providers regarding the risks associated with this intervention, ease of placement in urgent settings, or limited access to hepatology or interventional services. The continued use of ICT for HH despite its associated risks emphasizes the need for increased provider education and adherence to evidence-based management guidelines. To reduce inappropriate use, hospitals should implement standardized protocols, expand access to alternative therapies, and promote multidisciplinary care involving hepatology, pulmonology, and interventional radiology. At a systemic level, hospitals must implement protocols that discourage ICT placement in HH patients and promote the use of appropriate alternative therapies such as TIPS, ICT, or IPCs.

Although large sample size using NIS database is a strength of our study, we recognize certain limitations of our study. The retrospective study design and reliance on administrative data from the NIS database may introduce selection bias and coding inaccuracies. Furthermore, long-term outcomes outside of hospitalization have not been captured in our study, which would be important for assessing association of ICT with long-term patient survival and their quality of life. Interestingly, we found that a substantial proportion of patients (31%) without coded HH underwent thoracentesis. This likely reflects alternate etiologies of pleural effusion—such as parapneumonic effusion, fluid overload, or undiagnosed HH, underscoring the limitations of administrative coding. In addition, while propensity score matching was used, the retrospective nature of our analysis precludes insight into clinical reasoning for ICT placement, which may be influenced by factors such as provider judgment, hospital resources, and patient. Despite these limitations, our findings align with existing literature and offer compelling evidence against the frequent use of ICT in HH management.

In conclusion, our study demonstrates that ICT placement for HH is associated with increased morbidity, greater resource utilization, and higher IHM. Despite these risks, ICT use continues to rise, underscoring the need for increased awareness and adherence to alternative evidence-based management strategies. Future efforts should focus on educating providers, implementing hospital protocols to discourage ICT placement, and optimizing multidisciplinary care for patients with HH.

## CONFLICTS OF INTEREST

**Guarantor of the article:** Ashwani K. Singal, MD, MS, FACG.

**Specific author contributions:** Concept and design: A.T. and A.K.S. Experiments and procedures: A.K.S. Data interpretation: A.T., S.P., A.K.S. Writing the article draft: A.T. and S.P. wrote the first draft. All the authors reviewed, provided intellectual input, and approved the final draft.

**Potential competing interests:** A.K.S. reports (i) Speaker's Bureau and Writing for Medscape Gastroenterology, Chronic Liver Disease Foundation, Expert Perspectives, Gastro Endo News, Dynamed, Medical Education Speakers Network, Up-to-Date, Madrigal Pharmaceuticals; (ii) Grants to institution from ACG, NIH (NIAAA, NIDDK, and NIGMS), and DHHS; and (iii) Consultant on the SBIR grant for Pleiogenix pharmaceuticals.

**Financial support:** Kentucky Medicaid (SUP26-C5531), NIH (P20 GM103436 supplement), and NIAAA (U01 AA026980-06) to A.K.S.

**Study Highlights**

**WHAT IS KNOWN**

✓ Hepatic hydrothorax (HH) is a complication and a manifestation of decompensated cirrhosis. Intercostal chest tube (ICT) placement is discouraged because of associated complications.

**WHAT IS NEW HERE**

✓ Despite worse patient outcomes associated with ICT use including mortality and healthcare costs, the trend on use of ICT for the management of HH is increasing in hospitalized patients with cirrhosis.
✓ Clinicians should avoid ICT placement in HH when possible and consider safer, evidence-based alternatives such as repeated thoracentesis, TIPS, or indwelling pleural catheters.

## ABBREVIATIONS:

AKI: acute kidney injury
CI: confidence interval
HH: hepatic hydrothorax
ICD: International Classification of Diseases
ICT: intercostal chest tube
IHM: in-hospital mortality
LOS: length of stay
NIS: National Inpatient Sample
OR: odds ratio
SBE: spontaneous bacterial empyema
TC: total charges
TIPS: transjugular intrahepatic portosystemic shunt
